# Preparation of Chemically Resistant Cellulose Benzoate Hollow Fiber Membrane via Thermally Induced Phase Separation Method

**DOI:** 10.3390/membranes12121199

**Published:** 2022-11-28

**Authors:** Shota Takao, Saeid Rajabzadeh, Masahide Shibata, Chihiro Otsubo, Toyozo Hamada, Noriaki Kato, Keizo Nakagawa, Tooru Kitagawa, Hideto Matsuyama, Tomohisa Yoshioka

**Affiliations:** 1 Daicel Co., Ltd., 1239 Shinzaike, Aboshi-ku, Himeji 671-1283, Japan; 2 Graduate School of Science, Technology and Innovation, Kobe University, 1-1 Rokkodai, Nada, Kobe 657-8501, Japan; 3 Research Center for Membrane and Film Technology, Kobe University, 1-1 Rokkodai, Nada, Kobe 657-8501, Japan; 4 Department of Chemical Science and Engineering, Kobe University, 1-1 Rokkodai, Nada, Kobe 657-8501, Japan; 5 School of Civil and Environmental Engineering, University of Technology Sydney (UTS), City Campus, Broadway, Ultimo, NSW 2007, Australia

**Keywords:** cellulose acetate benzoate (CBzOH), hollow fiber membrane, microfiltration, thermally induced phase separation, chlorine resistance

## Abstract

For the first time, we have successfully fabricated microfiltration (MF) hollow fiber membranes by the thermally induced phase separation (TIPS) and non-solvent induced phase separation (NIPS) methods using cellulose acetate benzoate (CBzOH), which is a cellulose derivative with considerable chemical resistance. To obtain an appropriate CBzOH TIPS membrane, a comprehensive solvent screening was performed to choose the appropriate solvent to obtain a membrane with a porous structure. In parallel, the CBzOH membrane was prepared by the NIPS method to compare and evaluate the effect of membrane structure using the same polymer material. Prepared CBzOH membrane by TIPS method showed high porosity, pore size around 100 nm or larger and high pure water permeability (PWP) with slightly low rection performance compared to that by NIPS. On the contrary, CBzOH membranes prepared with the NIPS method showed three times lower PWP with higher rejection. The chemical resistance of the prepared CBzOH membranes was compared with that of cellulose triacetate (CTA) hollow fiber membrane, which is a typical cellulose derivative as a control membrane, using a 2000 ppm sodium hypochlorite (NaClO) solution. CBzOH membranes prepared with TIPS and NIPS methods showed considerable resistance against the NaClO solution regardless of the membrane structure, porosity and pore size. On the other hand, when the CTA membrane, as the control membrane, was subjected to the NaClO solution, membrane mechanical strength sharply decreased over the exposure time to NaClO. It is interesting that although the CBzOH TIPS membrane showed three times higher pure water permeability than other membranes with slightly lower rejection and considerably higher NaClO resistance, the mechanical strength of this membrane is more than two times higher than other membranes. While CBzOH samples showed no change in chemical structure and contact angle, CTA showed considerable change in chemical structure and a sharp decrease in contact angle after treatment with NaClO. Thus, CBzOH TIPS hollow fiber membrane is noticeably interesting considering membrane performance in terms of filtration performance, mechanical strength and chemical resistance on the cost of slightly losing rejection performance.

## 1. Introduction

Nowadays, the membrane separation method is considered one of the promising solutions to solve challenging global issues such as water scarcity, water pollution and global warming, mainly caused by population growth. Depending on the difference in the pore size of the membrane, several types of membrane are classified, such as microfiltration (MF), ultrafiltration (UF), nanofiltration (NF) and reverse osmosis (RO) membranes [1,2]. Among the different materials used for the preparation of the MF and UF membranes, polyvinylidene fluoride (PVDF) is an attractive material because it has high chemical and heat resistance. Nowadays, we can see PVDF membranes are widely used for potable water production plants worldwide. However, PVDF intensely suffers from fouling because it is a hydrophobic material property [3,4,5,6,7].

Contrary to the PVDF, cellulose acetate (CD) is hydrophilic and has a lower fouling tendency, so it is widely manufactured in RO, UF and MF membranes preparation [8,9,10,11,12,13]. Loeb and Sourirajan first applied CA to RO membrane separation in 1963 [14]. In addition, CA has been used as an MF or UF membrane for abatement of the water turbidity in water purification plants. However, contrary to the PVDF, it has much lower chemical resistance. Long-term exposure to sodium hypochlorite to CA reduced the strength of RO membranes and resulted in their breakage, causing membrane deterioration by reducing their salt rejection properties [15,16,17]. CA is susceptible to hydrolysis, which is mainly affected by the pH and temperature of the raw water. Due to that, the pH and operating temperature of the raw water are controlled within the 4.0–8.5 range, and the feed water temperature is kept below 45 °C [10,11,18,19]. To solve CA membrane poor chemical resistance, research is being conducted on developing new materials with excellent chlorine and alkali resistance by improving the primary CA material.

Only a few studies have focused on the chemical structural modification of the CA derivatives to make the membrane tolerable by sodium hypochlorite [17,20,21,22]. Arkhangelsky et al. [20,21] recognized that chemical cleaning of the cellulose acetate membrane with sodium hypochlorite induces the formation of the COOH, CHO and CO groups on the surface of the cellulose acetate membrane. In addition, Hashizume et al. [17] studied in detail the decomposition mechanism of CD by sodium hypochlorite and the enhancement of CD decomposition resistance during chemical washing. They used typical cellulose derivatives such as cellulose diacetate (CDA) and cellulose triacetate (CTA) to prepare filaments via non-solvent-induced phase separation (NIPS) methods [22,23]. In addition to CDA and CTA, filaments from cellulose acetate propionate (CDP) and cellulose acetate benzoate (CBzOH) were prepared to compare the resistance to sodium hypochlorite [17]. The filaments were immersed in 500 ppm and 2000 ppm sodium hypochlorite aqueous solution for 1–77 days. By evaluating the degree of substitution (DS) and molecular weight (Mw) of the cellulose derivative, it has been proposed that the degradation of CD by sodium hypochlorite has two possible mechanisms, including cleavage of the C1:C2 and C2:C3 bonds that can occur after de-esterification [17]. Moreover, by conducting a tensile strength test using filaments before and after the sodium hypochlorite test, the results showed various physical properties, such as tensile strength, elongation at break, and sodium chloride resistance, can be controlled by the introduction of aromatic bulky benzoyl groups to cellulose. They found that CD with the benzoyl group has an alkali hydrolysis rate about seven times slower than that of CD with the acetyl group [17].

Although it was shown that CBzOH is resistant to sodium hypochlorite, very few studies have used CBzOH for membrane preparation. Vyas et al. [24] evaluated the CBzOH flat sheet membrane structure used as the RO membrane by SEM. No other evaluation and characterization or comparison was made in this study. In the following work from the same group [25], the authors evaluated the preparation of the CBzOH RO flat sheet membrane prepared by the NIPS method and assessed the effect of casting solution composition and casting condition. The Prepared CBzOH RO flat sheet membrane was compared to a typical CTA membrane in salt water filtration performance. In none of the above-mentioned papers the resistance of CBzOH RO flat sheet membranes was evaluated against harsh chemicals such as NaClO.

In this study, for the first time, we have reported a method for producing porous MF hollow fiber membranes of CBzOH by TIPS and NIPS methods. Prepared CBzOH MF hollow fiber membrane by TIPS method was compared with those of CTA and CBzOH hollow fiber membranes prepared by NIPS method in terms of membrane structure, filtration performance, and especially chlorine resistance. It looks like membrane material and chemical structure, rather than membrane structure, play the key role in making a resistant membrane against chlorine. To the best of our knowledge, porous CBzOH membrane preparation has not been reported yet in any form of flat sheet or hollow fiber using any membrane preparation method, and only the NIPS method was used to prepare CBzOH RO flat sheet dense membranes [24,25].

## 2. Materials and Methods

### 2.1. Materials

**Figure 1:**
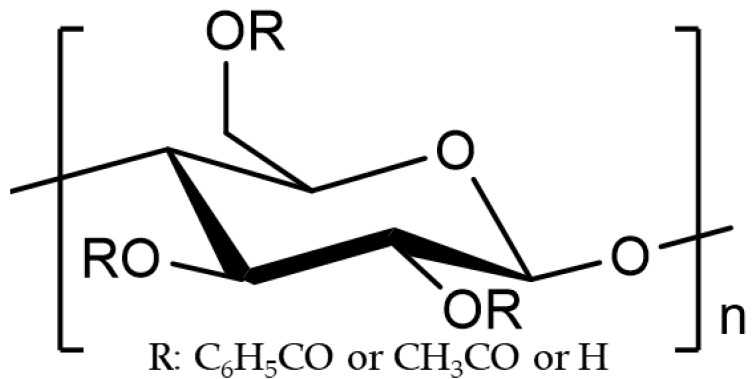
Chemical structure of the cellulose derivatives. CBzOH and CTA were used in this study with R groups which are listed in Table 1 [26].

In this study, CBzOH was used to prepare a hollow fiber membrane using both NIPS and TIPS methods after extensive solvent screening. Figure 1 shows the chemical structure of the cellulose derivative, and Table 1 shows the physical characteristics of the chemically modified-CA polymer, such as cellulose benzoate developed by Daicel (CBzOH: Mw578,846, Hyogo, Japan) and cellulose triacetate manufactured by Daicel (CTA: Mw405,000, Hyogo, Japan) that used to prepare the membrane. Sixty-one solvents were candidates in the TIPS process to dissolve CBzOH polymer with a boiling point of 180 °C or higher and a Hanssen solubility parameter (HSP) in the range of 8–34 [J/cm^3^], as summarized in Table A1. CTA was used to prepare the control hollow fiber membrane via NIPS method.

### 2.2. Solvent Screening

In order to prepare a homogenous polymer solution, 61 kinds of solvents were first selected based on the Hansen solubility parameter (HSP), as summarized and tabulated in Table A1. The HSP (*δt* [(J/cm^3^)^0.5^]) is the contribution of three types of interactions: dispersion (*δ_d_*), dipole interaction (*δ_p_*) and hydrogen bonding (*δ_h_*), as shown in Equation (1).

HSP = *δt* = (*δ_d_*^2^ + *δ_p_*^2^ + *δ_h_*^2^)^0.5^ (1)

The affinity between the polymer (component 1) and solvent (component 2) can be determined from the difference in their HSP difference values, which is denoted by Ra and expressed by Equation (2)

Ra = (4(*δ_d_*_1_ − *δ_d_*_2_)^2^ + (δ*_p_*_1_ − δ*_p_*_2_)^2^ + (*δ_h_*_1_ − *δ_h_*_2_)^2^)^0.5^ (2)

A smaller Ra value indicates a higher affinity between the polymer and the solvent. Generally, solvents appropriate for the TIPS process can dissolve the polymer at high temperatures, while at low temperatures, the solvent should not dissolve the polymer. Therefore, for a good candidate solvent in TIPS, the Ra value (the difference in HSP between the polymer and solvent) should be neither too small nor too large so that the dope solutions of polymer and solvent become homogeneous at the high temperature, undergo phase separation during the cooling process, and form porous structures by TIPS process.

In the solvent screening test, the polymer (0.3, 0.4 and 0.5 g) and solvent (1.7, 1.6 and 1.5 g) were weighed and put into a test tube to prepare polymer solutions at the concentrations of 15, 20 and 25 wt%, respectively. The mixture was heated on an aluminum block at 170 °C and stirred for 3 h to obtain a homogenous and transparent polymeric solution. Afterward, the test tube was placed in a holder and cooled to room temperature in the air. At this point, we checked whether the dope in the test tube became white and solidified or not. The first criterion used for solvent screening is that the system should appear as a homogenous polymeric solution at 170 °C, and in the second step of screening, we considered the polymeric solution solidifies at room temperature with considering an appropriate solidification rate. After the first screening, 42 solvents were selected (Table A2), and 8 solvents remained after the second screening (Table A3). For the selected dope solutions, the cloud points were measured by the method described in the following section.

### 2.3. Phase Separation Temperature Measurement

Similar to our previous study [26], for the cloud point measurement, in order to prevent the solvent from evaporating during heating, the sample obtained from the solvent screening in part 2.2 was sandwiched between two cover glasses and a Teflon sheet, and the gap was filled with grease. The sample was placed on a hot stage (Linkam, HFS91, Salfords, UK), increased the temperature to reach 190 °C and then kept at this temperature for 1 min and cooled to 25 °C at a rate of 10 °C/min. The cloud point temperature was visually observed by observing the state of turbidity with an optical microscope (Olympus, BX50, Tokyo, Japan). Approximately 6–8 mg of the sample prepared by the polymer and solvent screening shown in Table A3 was weighed, and the sample was placed in an aluminum pan. A differential scanning calorimeter (DSC Q2000, TA Instruments, New Castle, DE, USA) was put into this sample container. The melting point and crystallization temperature of the sample were measured under a nitrogen atmosphere by varying the temperature from 0 °C to 250 °C and cooling from 250 °C to 0 °C at a heating or cooling rate of 10 °C/min.

### 2.4. Preparation of Hollow Fiber Membrane

A continuous twin-screw extruder was used to prepare a hollow fiber membrane provided by the company (KURIMOTO, LTD., K.R.C. kneader, Osaka, Japan). Table 2 shows the conditions for producing CBzOH hollow fiber membrane by the TIPS process. 1,3-Butylene glycol (1,3-BG; Wako Pure Chemical Industries, Ltd., Osaka, Japan, special grade, ≥98.0%) was used as TIPS solvent for membrane preparation.

**Figure 2:**
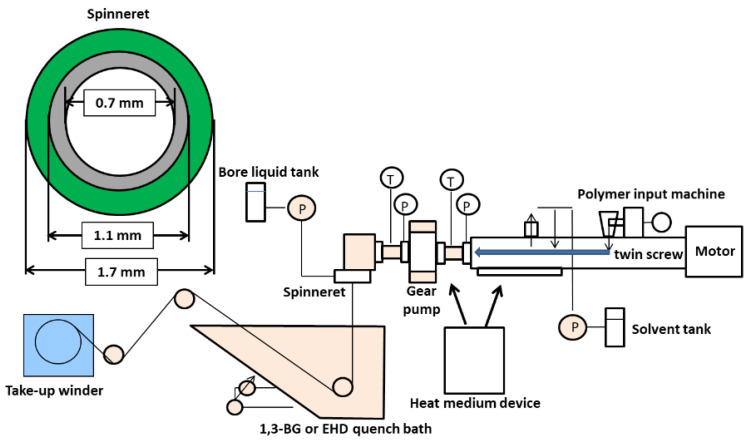
Schematic of the twin-screw kneader for hollow fiber membranes via the TIPS.

A hollow fiber membrane was prepared using the twin-screw extruder for the TIPS process shown in Figure 2. In brief, predetermined amounts of polymer (CBzOH) and solvent (1,3-BG) were fed to the polymer input machine and solvent tank. After that, the flow rates of the polymer and the solvent are controlled and supplied to the spinning nozzle from the twin-screw heated to 190 °C. Twin screw technology is very sophisticated in obtaining a homogenous polymeric solution. It is reported in several hundred papers make a polymeric solution to make a membrane. If the temperature of the twin-screw kneader is set to 190°C, the cellulose derivative will dissolve in the solvent in a short period of time, resulting in a homogeneous polymer solution. Furthermore, the obtained polymer solution was flown to the spinneret having a double channel structure by a gear pump, and at the same time, the solvent was flown to the inner channel of the spinneret as the bore liquid. The polymer solution was cooled in a solution (1,3-BG/water = 95 wt%/5 wt%) at 26 °C in the quench bath, and the solvent was exchanged with water to obtain a hollow fiber membrane. After washing the prepared hollow fiber membrane with water, the residual solvent in the membrane bulk was exchanged by sequentially immersing the prepared hollow fiber membrane in ethanol, diethyl ether and hexane for 30 min for each solvent. The prepared hollow fiber membrane was subjected to pure water permeability, a tensile test and a rejection measurement of 0.1 μm colloidal silica particles solution.

The reason that 1,3-BG was selected from all solvents for CBzOH was that the polymer dissolved uniformly at 190 °C and solidified rapidly at room temperature. Since the HSP of the 1,3-BG is much closer rather than that of the water, using 1,3-BG resulted in a more porous outer surface [26,27]. Thus, the membrane surface porosity became controllable when 1,3-BG was used as the quenching medium. However, CBzOH with 1,3-BG alone shows a very slow solidification rate and deforms the hollow fiber membrane shape. Therefore, by adding 5 wt% of water to 1,3-BG (1,3-BG/water = 95 wt%/5 wt%), while membrane outer surface porosity was kept high, a perfectly circular hollow fiber membrane was formed. Therefore, 1,3-BG/water = 95 wt%/5 wt% was used as the CBzOH quenching bath liquid.

In order to evaluate the effect of membrane structure on chlorine resistance, CBzOH MF hollow fiber membranes were prepared using the NIPS method. In order to compare our CBzOH-prepared membranes via TIPS and NIPS methods, we also designed a CTA control membrane with the NIPS method to evaluate the effect of the membrane chemical structure (membrane material). Detail of the CBzOH and CTA membrane preparation by NIPS and characterization are included in the Appendix A.2.

### 2.5. Evaluation of the Prepared Hollow Fiber Membrane

#### 2.5.1. SEM Observation

Membranes were air-dried for 1 h and then kept overnight in an oven at 55 °C. After fracturing the dry hollow fiber membranes in liquid nitrogen and sputtering with Pt, the cross-section, outer surface and inner surface of membranes were observed using field-emission scanning electron microscopy (FE-SEM, JEOL, JSF-7500F, Tokyo, Japan) at a scanning voltage of 3.0 kV.

#### 2.5.2. Pure Water Permeability (PWP)

The pure water permeability was evaluated using a sample immersed in ethanol for two weeks and then washed with running water for 30 min. Pure water permeability (PWP) through the hollow fiber membrane was measured by a method similar to that described in our previous work [26]. Pure water was forced to permeate from the inside to the outside of the hollow fiber membrane under a transmembrane pressure of 0.1 MPa. The inside-to-out permeability test was adopted to examine PWP for easy operation. The water permeability was calculated based on the inner surface area of the hollow fiber membrane.

#### 2.5.3. Particle Rejection

The 100 nm silica particle rejection experiment was performed by flowing the feed solution through the membranes’ outer surface (dense layer). Water permeated from the outer surface to the inner surface, similar to our previous works [26,28]. The feed solution was prepared by adding the silica particle (100 nm, Quarton@, PL-7 grade, Fuso Chemical Industry, Nagano, Japan) in the pure water (100 ppm colloidal silica aqueous solution). The filtrate and feed solution’s particle concentration was measured with the portable turbidity meter (HACH 2100P, Hach Co., Tokyo, Japan) with visible light in the 400–600 nm wavelength range. The particle rejection, R, was defined by Equation (3):

(3) $$R\;\%\;=\;(\frac{C_{0}-C_{f}}{Co})\;\times \;100$$

where *C*_0_ and *C_f_* are particle concentrations in the feed and permeate, respectively.

#### 2.5.4. Chlorine Resistance and Alkali Resistance Test

The NaClO aqueous solution used for the chlorine resistance test was prepared as follows. Pure water was added to 13.5 g of the undiluted solution of NaClO aqueous solution (Shoukou Kasei Co., Ltd., tokyo, japan, Hisicrine S, ≥12.0%) to make a total of 1000 g solution, and the mixture was stirred. Using a chlorine meter quality meter (AQABU, AQ-202 type, Shibata Scientific Technology, Tokyo, Japan), it was confirmed that the effective chlorine concentration was 2000 ppm with pH 12. Next, the prepared hollow fiber membrane was washed with running water for 30 min and immersed in the prepared NaClO aqueous solution for 7, 10 and 14 days. After that, the mechanical strength was measured using a tensile strength measurement machine (EZ-SX, Shimadzu Corporation, Kyoto, Japan) for fresh membranes and membranes immersed in NaClO solution for different time intervals. To investigate the effect of the NaClO treatment on the CTA and CBzOH samples, the attenuated total reflection Fourier transform infrared (ATR-FTIR) was carried out using Alpha Bruker. The samples’ water contact angle was measured using a goniometer (Drop Master, Kyowa Interface Science Co., Saitama, Japan).

## 3. Results

### 3.1. Solvent Screening for Membrane Preparation via TIPS

The two steps solvent screening method reported in our previously published paper [26] and described briefly in Section 2.2 was carried out for CBzOH polymer solvent screening. In this experimental evaluation, we can see and find the appropriate solubility, viscosity, solidification rate and processability of the polymeric solution. We think that for the aim and scope of our study, it is acceptable to use HSP rather than interaction parameters. As shown in Table A2, the number of solvents appropriate for CBzOH dissolution was 42 in the Ra range of 8.7 to 14.9 [(J/cm^3^)^0.5^]. After considering the processability of selected solvents (the second criterion of screening), eight solvents were found suitable for CBzOH with Ra in the range of 8.7–12.4 [(J/cm^3^)^0.5^]. These results are shown in Table A3. Finally, we selected 1,3-BG as the solvent for preparing the CBzOH hollow fiber membranes based on the HSP evaluation, acceptable viscosity, solidification of polymer solutions at room temperature, and good mechanical strength after solidification. The HSP of the polymer CBzOH and the selected solvent 1,3-BG are summarized in Table A4.

### 3.2. Phase Diagram of CBzOH/1,3-BG for the TIPS

**Figure 3:**
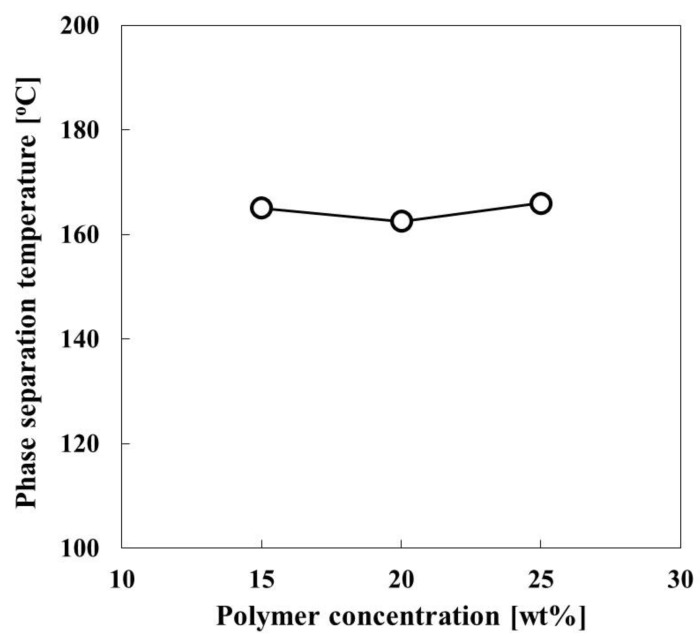
Phase diagram of CBzOH/1,3-BG solutions.

Figure 3 shows the phase diagram of the CBzOH/1,3-BG system. The cloud points of polymer solutions with polymer concentrations of 15, 20 and 25 wt% are around 165 °C. The crystallization temperature of CBzOH/1,3-BG could not be confirmed by DSC measurement. Thus, a liquid-liquid phase separation mechanism is expected to accomplish the membrane formation, and the membrane structure will be interconnected. Although in most studies, crystallization was observed for cellulose derivatives in the published papers [29,30,31,32,33,34,35,36], no crystallization was observed in this study. Considering the Ra of the CBzOH/1,3-BG is 12.4 (Table A2) [(J/cm^3^)^0.5^], the compatibility of the CBzOH with 1,3-BG may not be high enough to observe the crystallization temperature.

### 3.3. Membrane Structure and Performance

#### 3.3.1. Membrane Structure

**Figure 4:**
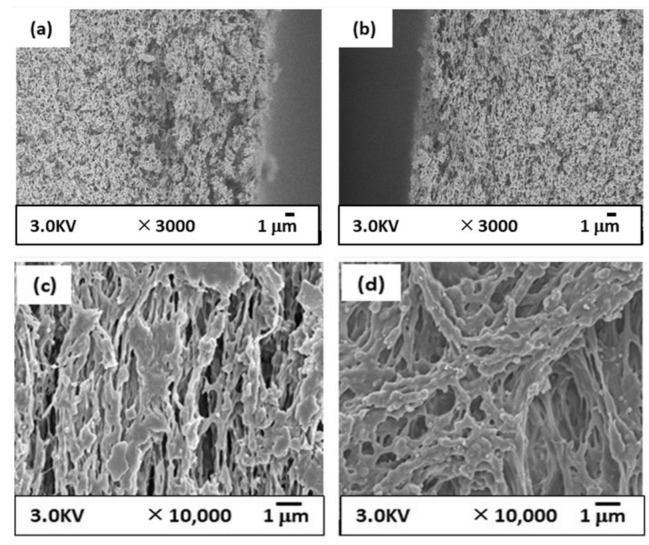
SEM images of prepared CBzOH hollow fiber membranes prepared by TIPS method; (**a**): cross-section near the outer surface (**b**): cross-section near the inner (**c**) outer surface (**d**) inner surface.

Figure 4 shows the structure of the prepared CBzOH membrane via the TIPS method. From the cross-section structure, Figure 4a,b, it is clear that the membrane structure is an entirely porous interconnected structure with a pore size of approximately 100 nm or larger. This structure is expected considering that phase separation takes place by liquid-liquid phase separation without any crystallization, as explained in Section 3.2. This kind of structure is not a typical structure because, in most studies, the spherical structure was observed for membranes prepared by cellulose derivatives [29,30,31,32,33,34,35,36]. It is clear from the SEM images of the outer and inner surfaces of the CBzOH membrane (Figure 4c,d) that membrane surfaces are entirely porous with a pore size diameter of around 100 nm or larger. Usually, in the TIPS process, the membrane’s outer surface is dense due to solvent evaporation during the air gap distance [37]. In this study, 1,3-BG/water = 95 wt%/5 wt% is used as a solvent, and the solvent used in membrane polymer solution preparation is 1,3-BG. Thus, the membrane’s outer surface is very porous [38].

**Figure 5:**
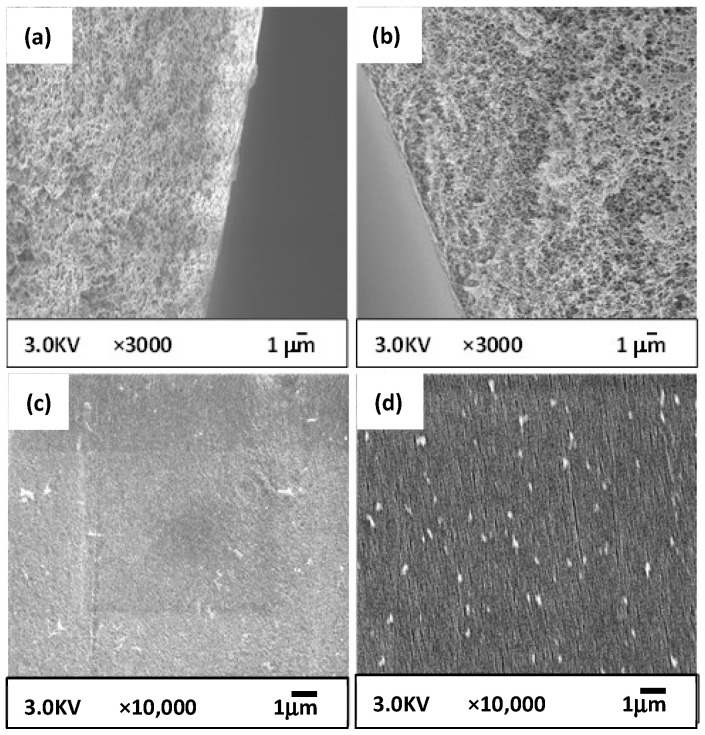
SEM images of prepared CBzOH hollow fiber membranes prepared by NIPS method; (**a**): cross-section near the outer surface (**b**): cross-section near the inner (**c**) outer surface (**d**) inner surface.

As explained in Section 2.4, the CBzOH NIPS membrane was prepared to evaluate the net effect of the membrane structure using the same polymer because we strongly believe by using the TIPS and NIPS methods, two utterly different structures with different mechanisms is formed. Figure 5 shows the structure of the prepared CBzOH membrane via the NIPS method. Overall, it is completely clear that although the same material (CBzOH) was used for membrane preparation, using TIPS and NIPS methods, we obtained different structures by comparing Figure 4 with Figure 5. Comparing the cross-section structure of the CBzOH NIPS membrane (Figure 5a,b) with those of the TIPS structure (Figure 4a,b), the prepared CBzOH NIPS membrane shows a somehow denser structure rather than that of the TIPS structure, especially if the cross-section near the outer surface is compared. From the cross-section structure shown in Figure 5a,b, it is clear that the membrane structure is an entirely sponge-like structure. Contrary to the CBzOH TIPS membranes in that the inner and outer surface structures are very porous (Figure 4c,d), the inner and outer surface structures of CBzOH NIPS membranes are much denser, as shown in Figure 5c,d. Tiny pores with diameters less than 50 nm were observed at the inner and outer surfaces of the membrane. As the coagulation bath is only water, we can expect a dense structure at the outer and inner surfaces of the membrane.

**Figure 6:**
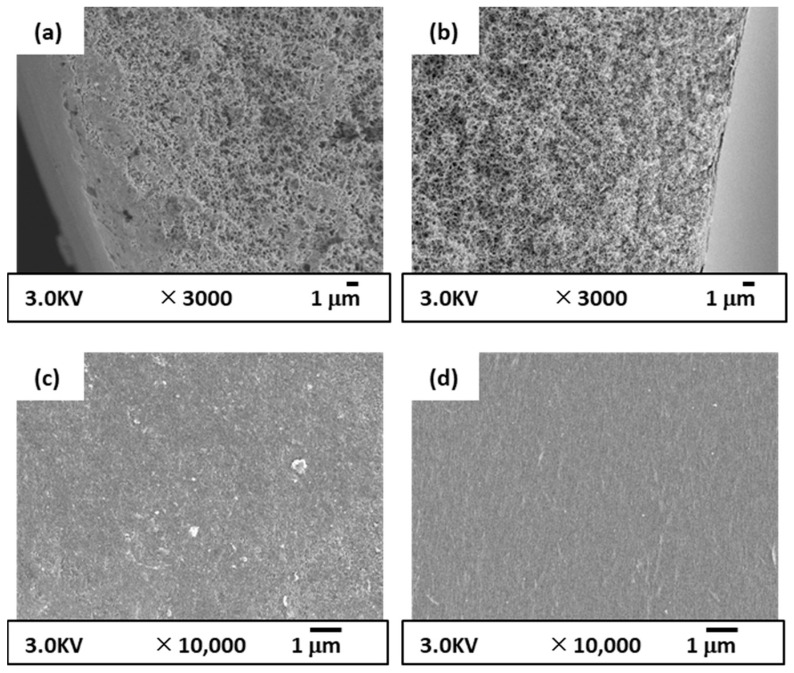
SEM images of prepared CTA hollow fiber membranes prepared by NIPS method; (**a**): cross-section near the outer surface (**b**): cross-section near the inner (**c**) outer surface (**d**) inner surface.

As explained in Section 2.4, CTA hollow fiber membrane was prepared in parallel to CBzOH TIPS and NIS membrane to evaluate the net effect of the membrane material. The structure of the prepared CTA membrane via the NIPS method is shown in Figure 6. Cross-section images (Figure 6a,b) show a complete sponge structure without any finger-like macrovoid formation. Figure 6a shows a somehow dense near-outer surface structure with a porous underneath membrane. On the other hand, the cross-section near the inner surface of the membrane structure is entirely porous. This difference at the cross section near the outer and inner surface is related to the air gap distance that some solvents evaporate from the outer surface of the membrane and make a skinny dense layer near the outer surface. Very similar to the CBzOH membrane prepared by the NIPS method, the inner surface and outer surfaces are dense with tiny pores around 50 nm.

As a conclusion of the SEM images (Figure 4, Figure 5 and Figure 6), when the TIPS method was applied for CBzOH hollow fiber membrane preparation with the condition mentioned before, completely porous MF membranes with a pore size of 100 nm or larger were obtained. On the contrary, when the NIPS method was applied for CBzOH or CTA, the membrane structure was denser, with pores smaller than that of the bulk membrane. From the cross-section structure, no spherulitic structure was observed in any prepared membrane, and just an interconnected structure was observed for all membranes at the bulk of the membrane.

#### 3.3.2. Pure Water Permeability (PWP) and Particle Rejection

**Figure 7:**
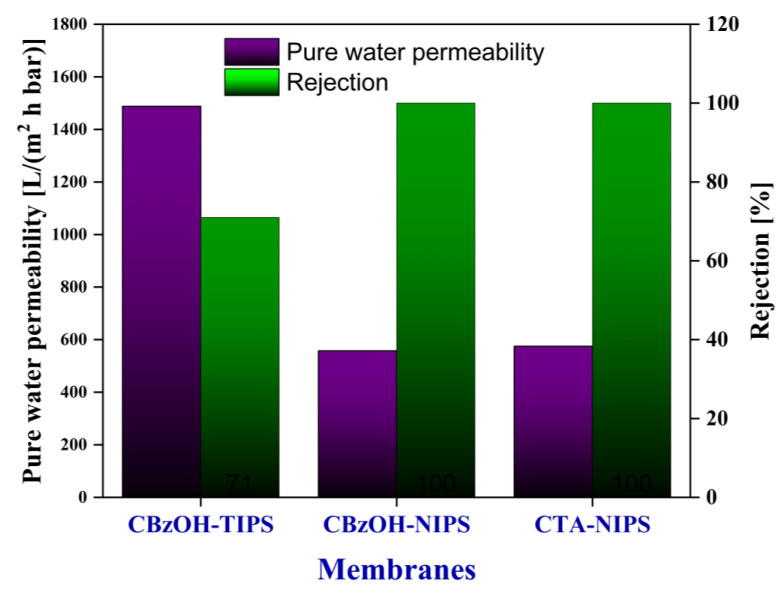
Pure water permeability and rejection of the membranes.

PWP and rejection of the prepared hollow fiber membranes were measured, and the results are summarized in Figure 7. As it is clear from Figure 7, while the PWP of the CbzOH TIPS hollow fiber membrane was around 1500 L/(m^2^ h bar) with silica particle rejection around 70%, the prepared hollow fiber membranes with NIPS process showed much lower PWP around 600 L/(m^2^ h bar) and 100% rejection of silica particles. These results are completely in line with SEM images. As explained in SEM images, the TIPS-prepared membrane was much more porous with a large pore size, resulting in higher PWP and low silica particle rejection. On the contrary, the NIPS-prepared membranes showed almost one-third of the PWP of the TIPS membrane with 100% rejection of the silica particles.

In general, the mean pore size measurement and solution rejection test are the consolidated criteria to determine the membrane type. Since CBzOH-TIPS membrane rejection for 100 nm silica particles is less than 90%, it falls in the range of the MF membrane based on the basic definition of the MF membranes [2]. For CBzOH-NIPS, the rejection for 100 nm silica particles is 100% which means even minimum pore sizes are smaller than 100 nm, and it does not pass any 100 nm silica particles at all. Thus, CBzOH-NIPS membranes fall in the range of the UF membranes range based on the definition [2].

Considering SEM images (Figure 4, Figure 5 and Figure 6) and PWP and rejection results (Figure 7), it can be concluded that the CBzOH TIPS membrane in the MF membrane range and the prepared NIPS membranes (CBzOH and CTA) are UF-type membranes.

#### 3.3.3. Chlorine Resistance

**Figure 8:**
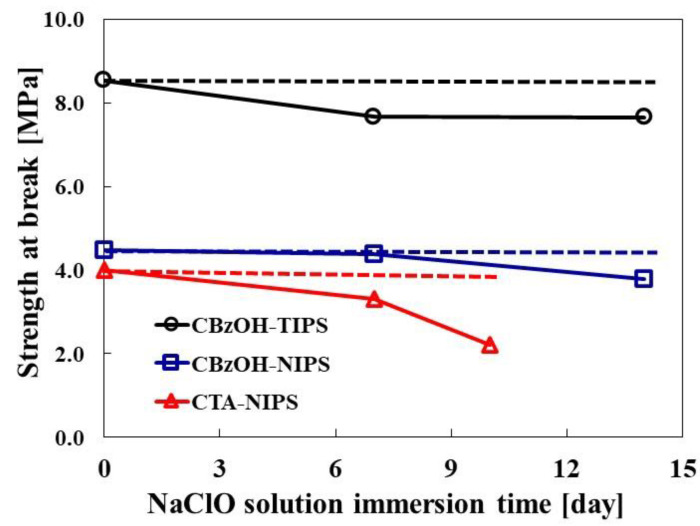
Immersion test of cellulose derivative in NaClO aqueous solution. (NaClO concentration: 2000 ppm).

Three types of membranes prepared from cellulose derivatives were immersed in an aqueous NaClO solution with a concentration of 2000 ppm to evaluate the chemical resistance of the membranes. The results of membrane chemical resistance against the NaClO are summarized in Figure 8. From Figure 8, the strength at the break after 14 days of immersion in the CBzOH TIPS membrane in the NaClO aqueous solution decreased slightly from 8.5 MPa to 7.7 MPa, and for the CBzOH NIP membrane, it decreased slightly from 4.5 MPa to 3.8 MPa. Both prepared membranes retain the initial mechanical strength after two weeks of immersing membranes for more than 85%, which means CBzOH material is very strong against the NaClO solution regardless of the membrane structure since for both TIPS and NIPS membranes they retain the initial membrane mechanical strength high enough. The difference in the initial strength of the TIPS and NIPS membranes comes from the different structures of the TIPS and NIPS membranes; generally, TIPS membranes have much higher mechanical strength than the NIPS membrane [1,2,39,40]. Although we firmly believe that the entirely different phase separation mechanisms of the CBzOH membranes in TIPS and NIPS membranes are the main reason for the different initial mechanical strength of these two membranes, the higher polymer concentration of the TIPS membrane (22%) rather than NIPS membrane (14%) might be another reason that resulted in much higher mechanical strength of the CBzOH TIPS membrane than that of the NIPS membrane.

**Figure A2:**
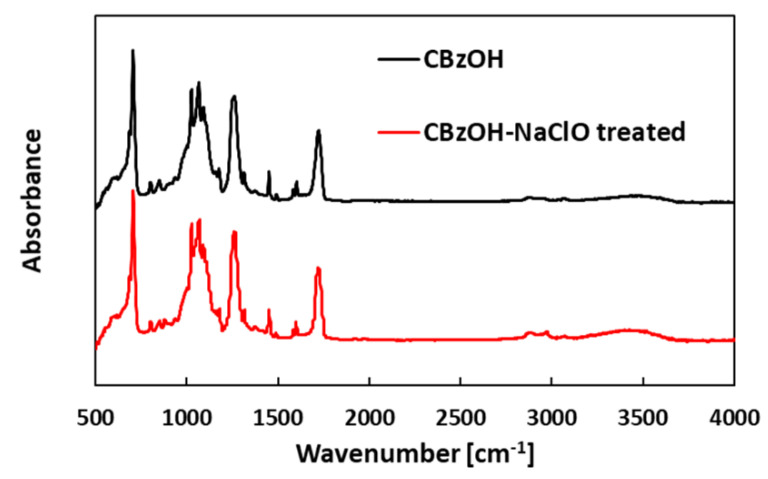
FT−IR of CBzOH samples before and after sodium hypochlorite immersion. Samples are immersed in sodium hypochlorite solution with 2000 ppm concentration for 7 days.

**Figure A3:**
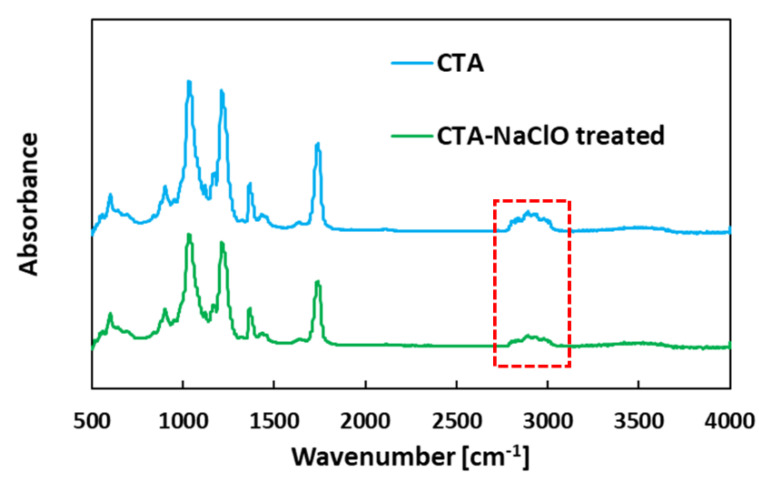
FT−IR of CTA samples before and after sodium hypochlorite immersion. Samples are immersed in sodium hypochlorite solution with 2000 ppm concentration for 7 days.

Contrary to the CBzOH membranes, the chemical strength of the CTA control membrane against the NaClO is entirely different. The strength at break of the CTA-NIPS membrane after 10 days of immersion in NaClO aqueous solution decreased sharply from 4.0 MPa to 2.2 MPa, which means strength retention was 55%. From the results in Figure 8, it can strongly be claimed that regardless of the membrane structure and formation mechanism, the CBzOH membrane has improved chlorine resistance. This is because the benzoyl group, which is bulkier than the acetyl group, in the cellulose backbone prevents chlorine radicals from attacking the cellulose chain [10]. To further evaluate the effect of the NaClO treatment on the chemical structure of the samples, FTIR (Figure A2 and Figure A3) and water contact angle (A6) evaluations were performed. As it is explained well with the results in Appendix A, FTIR and water contact angle assessment results strongly claim that while CTA is extremely vulnerable to NaClO, change in CBzOH samples is marginal. That proves consolidated that the benzoyl group prevents chlorine radicals attack.

## 4. Conclusions

We have successfully fabricated CBzOH porous hollow fiber membranes by the thermally induced phase separation (TIPS) method and non-solvent induced phase separation (NIPS) methods for the first time. For CBzOH TIPS membrane preparation, a comprehensive solvent screening was performed to obtain an appropriate TIPS solvent for CBzOH. HSP and solubility of the CBzOH at high temperatures were considered as the first criteria for screening solvent, and in the second step, the processability of the prepared polymeric solution for hollow fiber membrane preparation was considered. CTA hollow fiber membrane was also prepared via the NIPS method as the control membrane, which is the most typical product from the cellulose derivatives group. Prepared CBzOH with the TIPS method showed a completely interconnected structure with high porosity, a large pore size of 100 nm or larger, high pure water permeability (PWP) of 1500 L/(m^2^ h bar) with slightly low rejection around 70% for silica particles. On the contrary, CBzOH and CTA membranes prepared with the NIPS method showed three times lower PWP with 100% rejection of the silica particles. The chemical resistance of the prepared CBzOH membranes against NaClO 2000 ppm concentration solution was compared with that of the CTA hollow fiber membrane. CBzOH membranes prepared with TIPS and NIPS methods showed noticeably high resistance against the NaClO solution over two week’s immersion in 2000 ppm NaClO solution regardless of the membrane structure, porosity and pore size. On the contrary, the mechanical strength of the CTA membrane sharply decreased over the exposure time to NaClO. CBzOH TIPS hollow fiber membrane is noticeably interesting over other membranes, CBzOH NIPS and CTA NIPS, considering filtration performance, mechanical strength and chemical resistance on the cost of slightly losing rejection performance. Using FTIR and water contact angle, it was confirmed that while the CTA samples chemical structure was strongly affected by NaClO treatment and resulted in a sharp decrease in water contact angle, the change in the chemical structure of the CBzOH sample after NaClO treatment was marginal and subsequently the water contact almost remain almost intact.

## Figures and Tables

**Table 1:** Properties of the used.

Polymer	Tm *^1^ [°C]	Tg *^2^ [°C]	MW	Substitution Ratio *^3^		
				R:C_6_H_5_CO	R:CH_3_CO	R:H
CBzOH	-	196	578,846	2.1	-	0.9
CTA	300	-	405,000	-	2.87	0.13

*1: Melting point, *2: Glass-transition temperature, *3: Ratio of the esterified groups of 3 hydroxy groups per glucose unit [14].

**Table 2:** Preparation Conditions of CBzOH Hollow Fiber Membranes via TIPS method.

Preparation Conditions		Parameters
Polymer solution composition [wt%]	CBzOH/1,3-BG	22/78
Screw temperature [°C]		189 °C
Screw speed [rpm]		52 rpm
Polymer solution extruded rate [g/min]		28 g/min
Bore liquid		1,3-BG
Bore liquid flow rate [g/min]		14 g/min
Air gap [mm]		0
Quenching bath liquid		1,3-BG/water = 95 wt%/5 wt%
Quenching bath temperature [°C]		26 °C
Take-up speed [m/min]		30 g/min

**Figure A1:**
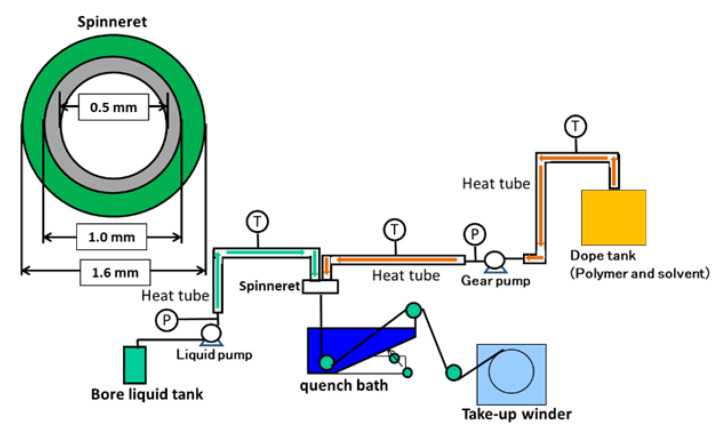
Schematic of the hollow fiber membrane preparation via the NIPS process.

## Data Availability

The data presented in this study are available on request from the corresponding author.

## Appendix A

### Appendix A.1. Solvent Screening

Table A1 shows the Hansen solubility parameter (HSP) of polymers and 61 types of solvents used in the solvent screening. Used polymers were two types of cellulose derivatives: cellulose benzoate developed by Daicel (CBzOH: Mw578,846, Hyogo, Japan), and cellulose triacetate manufactured by Daicel (CTA: Mw405,000, Hyogo, Japan). In addition, used solvents were Glycerin (Nacalai Tesque, inc., special grade, ≥99.5%, Kyoto, Japan), 1,5-pentanediol (Tokyo Chemical Industry, >97.0%, Tokyo, Japan), neopentyl glycol (NPG) (Tokyo Chemical Industry, >98.0%, Tokyo, Japan), 2-Methyl-2,4-pentanediol (Wako Pure Chemical Industries, ≥99.0%, Osaka, Japan), Diethylene glycol (Wako Pure Chemical Industries, ≥99.0%, Osaka, Japan), 1,3-butylene glycol (1,3-BG) (Wako Pure Chemical Industries, Ltd., special grade, ≥98.0%, Osaka, Japan), Tetraethyl glycol (Wako Pure Chemical Industries, Ltd., special grade, ≥95.0%, Osaka, Japan), Tripropylene glycol (Wako Pure Chemical Industries, Ltd., special grade, ≥97.0%, Osaka, Japan), Triethylene glycol (Tokyo Chemical Industry, >99.0%, Tokyo, Japan), N-Ethyltoluene Sulfonamide (Tokyo Chemical Industry, >98.0%, Tokyo, Japan), Diethylene glycol monoacetate (Tokyo Chemical Industry, >98.0%, Tokyo, Japan), 2-ethyl-1,3-hexanediol (EHD) (Wako Pure Chemical Industries, Ltd., ≥98.0%, Osaka, Japan), Dimethyl sulfoxide (Nacalai Tesque, inc., special grade, ≥98.0%, Kyoto, Japan), sulfolane (SF) (Wako Pure Chemical Industries, Ltd., ≥95.0%, Osaka, Japan), Dipropylene glycol (Wako Pure Chemical Industries, Ltd., ≥95.0%, Osaka, Japan), 2,5-dimethyl-2,5-hexanediol (Tokyo Chemical Industry, >99.0%, Tokyo, Japan), α-Butyrolactone (Tokyo Chemical Industry, >99.0%, Tokyo, Japan), Dialyl phthalate (Wako Pure Chemical Industries, Ltd., ≥98.0%, Osaka, Japan), α-terpineol (Tokyo Chemical Industry, >80.0%, Tokyo, Japan), 1,6-Hexanediol (Tokyo Chemical Industry, >97.0%, Tokyo, Japan), Tetrahydrofurfuryl acetate (Tokyo Chemical Industry, >97.0%, Tokyo, Japan), Bis phthalate (2-methoxyethyl) (Wako Pure Chemical Industries, Ltd., ≥96.0%, Osaka, Japan), Diethyl maleate (Tokyo Chemical Industry, >90.0%, Tokyo, Japan), Diethyl fumarate (Tokyo Chemical Industry, >98.0%, Tokyo, Japan), Mentanol (Wako Pure Chemical Industries, Ltd., ≥97.0%, Osaka, Japan), Propylene glycol diacetate (Wako Pure Chemical Industries, Ltd., ≥97.0%, Osaka, Japan), 1,4-Butanediol diacetate (Daicel Co., ≥98.0%, Hyogo, Japan), 1,3-butylene glycol diacetate (Daicel Co., ≥98.0%, Hyogo, Japan), Ethyl acetate (Daicel Co., ≥98.0%, Hyogo, Japan), Tarpinyl acetate (Tokyo Chemical Industry, >85.0%, Tokyo, Japan), Polyethylene glycol(6000) (Nacalai Tesque, inc., special grade, 100%, Kyoto, Japan), Tripropylene glycol-methyl-n-propyl ether (Daicel Co., ≥98.0%, Hyogo, Japan), Diisobutyl fumarate (Wako Pure Chemical Industries, Ltd., ≥98.0%, Osaka, Japan), Dihydrotestosterone acetate (Nippon Terpene Chemicals Co., ≥95.0%, Hyogo, Japan), Dipropylene glycol methyl-n-propyl ether (Daicel Co., ≥98.0%, Hyogo, Japan), Dipropylene glycol-methyl-isopentyl ether (Daicel Co., ≥98.0%, Hyogo, Japan), Triethyl phosphate (Tokyo Chemical Industry, >99.0%, Tokyo, Japan), Dimethyl phthalate (Wako Pure Chemical Industries, Ltd., ≥97.0%, Osaka, Japan), 1,1,3,3-tetramethylurea (Wako Pure Chemical Industries, Ltd., ≥98.0%, Osaka, Japan), Trimethyl phosphate (Wako Pure Chemical Industries, Ltd., ≥99.0%, Osaka, Japan), Triethyl citrate (Tokyo Chemical Industry, >98.0%, Tokyo, Japan), Dipropylene glycol methyl ether (Tokyo Chemical Industry, >98.0%, Tokyo, Japan), Dipropylene glycol n-propyl ether (Wako Pure Chemical Industries, Ltd., ≥95.0%, Osaka, Japan), Diethyl phthalate (Wako Pure Chemical Industries, Ltd., ≥98.0%, Osaka, Japan), Di-n-butyl phthalate (Tokyo Chemical Industry, >97.0%, Tokyo, Japan), Diethylene glycol monobutyl ether (Tokyo Chemical Industry, >99.0%, Tokyo, Japan), Dipropylene glycol n-butyl ether (Daicel Co., ≥98.5%, Hyogo, Japan), Dimethyl succinate (Tokyo Chemical Industry, >98.0%, Tokyo, Japan), Dimethyl adipate (Tokyo Chemical Industry, >99.0%, Tokyo, Japan), Diethyl succinate (Wako Pure Chemical Industries, Ltd., ≥97.0%, Osaka, Japan), Glycerol triacetate(Wako Pure Chemical Industries, Ltd., ≥99.0%, Osaka, Japan), Tripropylene glycol methyl ether (Tokyo Chemical Industry, >93.0%, Tokyo, Japan), Bis sebacate (2-ethylhexyl) (Tokyo Chemical Industry, >98.0%, Tokyo, Japan), Diethyl adipic acid (Tokyo Chemical Industry, >99.0%, Tokyo, Japan), o-Triethyl Acetyl Citrate (Tokyo Chemical Industry, >97.0%, Tokyo, Japan), Dipropylene glycol methyl ether acetate (Daicel Co., ≥98.0%, Japan), Bis phthalate (2-ethylhexyl) (Tokyo Chemical Industry, >98.0%, Tokyo, Japan), Di-n-butyl fumarate (Wako Pure Chemical Industries, Ltd., ≥98.0%, Osaka, Japan), Tributyl phosphate (Tokyo Chemical Industry, >99.0%, Tokyo, Japan), Di-n-butyl sebacate (Wako Pure Chemical Industries, Ltd., ≥97.0%, Osaka, Japan).

**Table A1:** Hansen solubility parameter (HSP) [27] and melting temperature of cellulose derivatives polymers and solvents used in the solvent screening.

Chemicals		*δd* [(J/cm^3^)^0.5^]	*δp* [(J/cm^3^)^0.5^]	*δh* [(J/cm^3^)^0.5^]	*δt* [(J / cm^3^)^0.5^]	Tm * [°C]
Polymer	Cellulose triacetate (CBzOH)	20.7	4.2	12.6	24.5	-
	Cellulose triacetate (CTA)	17.2	5.2	12.0	21.7	300
Solvent	Glycerin	17.4	11.3	27.2	34.2	18
	1,5-pentanediol	17.0	8.9	19.8	31.1	−16
	3-Methyl-1,5-pentanediol	16.7	8.1	17.6	29.5	No data
	Neopentyl glycol (NPG)	16.3	7.1	16.6	28.4	128
	2-Methyl-2,4-pentanediol	16.7	6.8	15.0	28.0	−40
	Diethylene glycol	16.6	12.0	19.0	27.9	−7
	1,3-butylene glycol (1,3-BG)	16.5	8.1	20.9	27.8	−77
	Tetraethyl glycol	16.5	9.4	15.3	27.8	−6
	Tripropylene glycol	16.9	9.9	13.9	27.6	−30
	Triethylene glycol	16	12.5	18.6	27.5	-
	N-Ethyltoluene Sulfonamide	18.6	13.0	7.8	27.4	-
	Diethylene glycol monoacetate	16.7	8.4	13.7	27.3	−32
	2-ethyl-1,3-hexanediol (EHD)	16.4	6.2	14.0	27.1	−40
	Dimethyl sulfoxide	18.4	16.4	10.2	26.7	18
	sulfolane (SF)	17.8	17.4	8.7	26.4	27
	Dipropylene glycol	16.5	10.6	17.7	26.4	−40
	2,5-dimethyl-2,5-hexanediol	16.4	5.6	11.0	25.7	89
	γ-Butyrolactone	18.0	16.6	7.4	25.6	−42
	Dialyl phthalate	17.8	8.5	4.0	25.5	-
	α-terpineol	17.1	3.6	7.6	25.3	31
	1,6-Hexanediol	15.7	8.4	17.8	25.2	39
	Tetrahydrofurfuryl acetate	16.9	6.5	7.2	25.0	-
	Bis phthalate (2-methoxyethyl)	17.2	9.7	5.4	24.9	−45
	Diethyl maleate	16.7	5.6	7.6	24.8	−10
	Diethyl fumarate	16.7	5.6	7.6	24.8	2
	Mentanol	16.8	3.5	6.6	24.7	35
	Propylene glycol diacetate	16.4	5.5	7.9	24.5	−75
	1,4-Butanediol diacetate	16.4	5.5	7.4	24.3	-
	1,3-butylene glycol diacetate	16.4	5.2	7.4	24.3	-
	Ethyl lactate acetate	16.3	5.5	7.6	24.3	-
	Tarpinyl acetate	16.7	2.8	4.2	24.0	<−80
	Polyethylene glycol(6000)	16.5	6.9	4.8	23.8	62
	Tripropylene glycol-methyl-n-propyl ether	15.9	6.2	7.1	23.6	-
	Diisobutyl fumarate	16.2	3.6	5.3	23.5	8
	Dihydrotestosterone acetate	16.4	2.7	3.4	23.4	-
	Dipropylene glycol methyl-n-propyl ether	15.6	4.3	4.1	22.4	−14.5
	Dipropylene glycol-methyl-isopentyl ether	15.5	3.7	3.7	22.2	-
	Triethyl phosphate	16.7	11.4	9.2	22.2	-
	Dimethyl phthalate	18.6	10.8	4.9	22.1	5.5
	1,1,3,3-tetramethylurea	16.7	8.2	11.0	21.6	-
	Trimethyl phosphate	15.7	10.5	10.2	21.5	−46
	Triethyl citrate	16.5	4.9	12.0	21.0	-45
	Dipropylene glycol methyl ether	15.5	5.7	11.2	20.0	−25.2
	Dipropylene glycol n-propyl ether	15.6	6.1	11.0	20.0	<−80
	Diethyl phthalate	17.6	9.6	4.5	20.5	−41
	Di-n-butyl phthalate	17.8	8.6	4.1	20.2	−35
	Diethylene glycol monobutyl ether	15.7	6.5	10.0	19.7	−68
	Dipropylene glycol n-butyl ether	15.7	6.5	10.0	19.7	<−75
	Dimethyl succinate	16.1	7.7	8.8	19.9	18
	Dimethyl adipate	16.3	6.8	8.5	19.6	10
	Diethyl succinate	16.2	6.8	8.7	19.6	-
	Glycerol triacetate	16.5	4.5	9.1	19.4	−78
	Tripropylene glycol methyl ether	15.3	5.5	10.4	19.3	<−78
	Bis sebacate (2-ethylhexyl)	16.2	5.0	9.0	19.2	−67
	Diethyl adipic acid	16.4	6.2	7.5	19.1	−20
	o-Triethyl Acetyl Citrate	16.6	3.5	8.6	19.0	-
	Dipropylene glycol methyl ether acetate	16.3	4.9	8.0	18.8	−25
	Bis phthalate (2-ethylhexyl)	16.6	7.0	3.1	18.3	−50
	Di-n-butyl fumarate	16.7	3.0	6.7	18.2	−35
	Tributyl phosphate	16.3	6.3	4.3	18.0	−79
	Di-n-butyl sebacate	16.7	4.5	4.1	17.8	−11

* DSC measured the melting temperature of polymers, and the melting temperature of the solvents were reported from the information provided by the supplier company.

**Table A2:** Solvents dissolved CBzOH polymer at 170 °C and Ra of the with the evaluated solvents (polymer concentration 25 wt%).

Solvent	Ra [(J/cm^3^)^0.5^]
1,5-pentanediol	10.2
Neopentyl glycol (NPG)	10.0
2-Methyl-2,4-pentanediol	8.7
Diethylene glycol	13.0
1,3-butylene glycol (1,3-BG)	12.4
Tetraethyl glycol	10.3
Tripropylene glycol	9.5
Triethylene glycol	13.9
N-Ethyltoluene Sulfonamide	10.8
Diethylene glycol monoacetate	9.0
2-ethyl-1,3-hexanediol (EHD)	8.9
Dimethyl sulfoxide	13.2
sulfolane (SF)	14.9
Dipropylene glycol	11.7
2,5-dimethyl-2,5-hexanediol	8.8
γ-Butyrolactone	14.4
Dialyl phthalate	11.1
α-terpineol	8.7
1,6-Hexanediol	12.0
Tetrahydrofurfuryl acetate	9.5
Diethyl maleate	9.4
Diethyl fumarate	9.4
Propylene glycol diacetate	9.8
1,4-Butanediol diacetate	10.0
1,3-butylene glycol diacetate	10.0
Ethyl lactate acetate	10.1
Triethyl phosphate	11.2
Dimethyl phthalate	10.9
1,1,3,3-tetramethylurea	9.0
Trimethyl phosphate	12.0
Dipropylene glycol methyl ether	10.5
Diethyl phthalate	11.5
Di-n-butyl phthalate	11.1
Diethylene glycol monobutyl ether	10.5
Dimethyl succinate	10.5
Dimethyl adipate	10.0
Diethyl succinate	10.1
Glycerol Triacetate (Triacetin)	9.0
Tripropylene glycol methyl ether	11.0
Diethyl adipic acid	10.1
o-Triethyl Acetyl Citrate	9.1
Tributyl phosphate	12.2

**Table A3:** Appropriate solvents for TIPS process and Ra of the CBzOH with the evaluated solvents (polymer concentration 25 wt%).

Solvent	Ra [(J/cm^3^)^0.5^]
3-methyl-1,5-pentanediol	10.2
2-Methyl-2,4-pentanediol	8.7
1,3-BG	12.4
Tripropylene glycol	9.5
EHD	8.9
Dipropylene glycol	11.7
2,5-dimethyl-2,5-hexanediol	8.8
α-terpineol	8.7

**Table A4:** The HSP of CBzOH and CTA polymer and selected solvent used in the membrane preparation by TIPS method [26].

		*δ_d_* [(J/cm^3^)^0.5^]	*δ_p_* [(J/cm^3^)^0.5^]	*δ_h_* [(J/cm^3^)^0.5^]	*δ_t_* [(J/cm^3^)^0.5^]	Tm * [°C]
Polymer						
	CBzOH	20.7	4.2	12.6	24.5	-
Solvent						
	1,3-BG	16.5	8.1	20.9	27.8	−77

* DSC measured the melting temperature of polymers, and the melting temperature of the solvents was reported from the information provided by the supplier company.

### Appendix A.2. Preparation of Hollow Fiber Membrane via Non-Solvent Induces Phase Separation (NIPS)

Table A5 shows the conditions for producing hollow fiber membranes of cellulose derivatives, and Figure A1 shows a schematic of the batch-type machine used for producing hollow fiber membranes by the NIPS process. Cellulose acetate benzoate (CBzOH) (Daicel, degree of benzoate substitution 2.1) and CTA were used as the polymer, and dimethyl sulfoxide (DMSO) (special grade, Wako Pure Chemical Industries, ≥95.0%, Osaka, Japan) was used as the solvent. Hollow fiber membranes were fabricated using the NIPS process equipment, was shown in Figure A1. Briefly, a dope tank is charged with a cellulose derivative (CBzOH or CTA), and a solvent (DMSO) and the tank temperature is set to 85–90 °C. Polymer dissolution and dope defoaming were performed overnight. The dope was supplied to the double tube spinneret by a gear pump, and at the same time, the temperature-controlled bore liquid was also supplied to the double tube spinneret by a pump. The dope and bore liquid were extruded from the double circular tube spinneret, passed through the air gap, and a quench bath to obtain a prepared hollow fiber membrane. The fabricated hollow fibre membrane was characterized in terms of pure water permeation rate, tensile test, and rejection of 0.1 µm colloidal silica particles.

**Table A5:** Preparation conditions of hollow fiber membranes by NIPS method.

Preparation Conditions		Parameters
Polymer solution composition [wt%]	CBzOH/DMSO	14/86
	CTA/DMSO	17/83
Dope tank temperature [°C]		85~90 °C
Gear pump temperature [°C]		90 °C
Dope solution flow rate [g/min]		14 g/min for CBzOH membrane
Bore liquid		Water
Bore liquid flow rate [g/min]		10 g/min for CBzOH membrane
Air gap [mm]		25
Quenching bath liquid		Water
Quenching bath temperature [°C]		90 °C
Take-up speed [m/min]		9 m/min for CBzOH membrane

Figure A2 and Figure A3 show the FT-IR observation results of the cellulose derivative sample before and after the sodium hypochlorite treatment. It is clear from the FTIR results in Figure A2, that there is no apparent difference for the CBzOH sample before and after treatment. Contrary to the CBzOH, as shown clearly in FTIR results in Figure A3, the intensity of the stretch peak at the range of 2850–2960 cm^−1^ attributed to the –CH_2_– group decreased after treatment. As it is explained well in the introduction part of our manuscript (Section 1, line 70–73) COOH, CHO and CO groups are formed after CDA and CTA exposure to hypochlorite, resulted in a decrease in –CH2– group peak intensity. Therefore, it can be concluded that while the CTA membrane is vulnerable to NaClO exposure and the chemical structure of the membrane polymer is altered seriously, the chemical structure of the CBzOH did not change after the NaClO treatment, and it is robust against corrosive chemicals such as NaClO.

Table A6 shows the water contact angles of the cellulose derivative samples before and after the sodium hypochlorite treatment. The water contact angles of CBzOH are about 76^°^ and 73^°^ before and after sodium hypochlorite treatment, respectively, which means sodium hypochlorite treatment had a mariginal effect on the water contact angle of the CBzOH samples. On the contrary to the CBzOH, water contact angles of CTA drop to 36^°^ from 58^°^ after sodium hypochlorite treatment. It indicates that sodium hypochlorite attacks the acetyl groups of CTA and changes it to hydroxyl groups that are more hydrophilic and resulted in a decrease in the water contact angle of the CTA after sodium hypochlorite treatment. Thus, it can be concluded that while the CTA chemical structure is vulnerable to sodium hypochlorite treatment and it changes the chemical structure of the CTA membranes, CBzOH samples are resistant to the sodium hypochlorite treatment, since the benzyl functional group prevents the sodium hypochlorite attack.

**Table A6:** Water Contact angles of cellulose derivative films before and after sodium hypochlorite treatment.

Membranes	Water Contact Angle (°)
CBzOH	76.2 ± 0.8
CBzOH NaClO treated	73.2 ± 0.8
CTA	58.4 ± 0.7
CTA NaClO treated	35.9 ± 0.5

Samples are immersed in sodium hypochlorite solution with 2000 ppm concentration for 7 days.
